# Integrative Morphological and Palynological Characterization of *Cocos nucifera* L.: Insights from Macro-Traits and Pollen Ultrastructure

**DOI:** 10.3390/plants15182860

**Published:** 2026-09-18

**Authors:** Chandrasekhar Manikala, Pijug Summpunn, Thanet Khomphet, Erfan Dani Septia

**Affiliations:** 1 School of Agricultural Technology and Food Industry, Walailak University, Nakhon Si Thammarat 80160, Thailand; chandrasekharmanikala003@gmail.com; 2 Food Technology and Innovation Research Center of Excellence, School of Agricultural Technology and Food Industry, Walailak University, Nakhon Si Thammarat 80160, Thailand; pijug.su@wu.ac.th; 3 Department of Agrotechnology, Faculty of Agriculture and Animal Science, University of Muhammadiyah Malang, Malang 65145, Indonesia; erfandani@umm.ac.id

**Keywords:** aromatic coconut, exine ornamentation, multivariate analysis, palynology, pollen morphometry, scanning electron microscopy

## Abstract

Coconut is popularly known as the “Tree of Life,” yet precise germplasm characterization of aromatic varieties remains challenging due to the high phenotypic plasticity of vegetative and fruit traits. This study evaluated phenotypic and palynological variation among ten coconut varieties using an integrated multidimensional morphometric (MIM) approach that combined morpho-agronomic traits with scanning electron microscopy (SEM)-based pollen observations and multivariate analyses. Analysis of variance and genetic variability analysis indicated substantial variation in plant height, stem girth, and especially fruit weight (H_b_^2^ = 0.97, GAM = 53.03%), suggesting that fruit weight may be a useful trait for further evaluation and selection within the studied collection. Pollen length (PL), width (PW), and pollen width-to-length ratio (PW/PL) did not differ significantly among varieties, indicating relatively limited variation in pollen dimensions within this collection. Polar length ranged from 33.82 to 44.55 μm, while the pollen width-to-length ratio (PW/PL), ranged from 1.06 to 1.37. Different pollen shapes, including navicular, reniform, ovoid, spherical, and subglobose forms, were observed within the examined samples. Multivariate analyses revealed variation among the varieties at both morphological and pollen levels. The agro-morphological-based PCA explained 74.4% of the total variation in the first two components and separated Var 10, Var 4, and Var 5 from several other varieties based on their combinations of vegetative, reproductive, and fruit-related traits. PCA based on pollen morphometrics explained 99.8% of the variation in the first two components, with pollen width, size index, and pollen length contributing strongly to varietal separation, particularly for Var 9 and Var 7. Qualitative pollen morphotype ordination further indicated differences in the distribution of pollen shape categories among varieties, while hierarchical clustering grouped varieties according to similarities in pollen dimensions and ratios, with Var 10 and Var 5 forming a group associated with relatively higher pollen width and size index values. These findings demonstrate that integrating morpho-agronomic and pollen-related characteristics can provide complementary information for characterizing variation within the evaluated coconut collection.

## 1. Introduction

*Cocos nucifera* L., commonly known as the coconut palm, is a perennial monocotyledonous species of enormous global socio-economic significance. It is often proclaimed as the “Tree of Life” and it sustains the livelihoods of more than 10 million smallholder farmers in 90 countries, and serves as important sources of food, fuel, and pharmaceutical raw materials [1]. Global coconut production reached approximately 68.8 billion nuts from 12.08 million hectares in recent estimates [1,2,3]. In recent years, the world market has experienced a surge in demand for high-value byproducts such as virgin coconut oil (VCO) and tender coconut water, which has driven the need to identify and develop better and more efficient cultivars [3]. However, the genetic enhancement and the systematic classification of the coconut is hampered because it has a long generation time, huge seed size and the complex nature of its reproductive biology [4]. Therefore, precise germplasm characterization is one of the key requirements for effective breeding and conservation strategies [5].

Traditionally, the classification of coconut types has been based on stature (tall vs. dwarf) and reproductive behavior (allogamous vs. autogamous) [6,7]. Morphological characterization, focusing on leaf, stem, and nut traits, has been the primary analytical tool for researchers. Leaf morphometrics, such as (leaflet count and length of the petiole) provides information in relation to photosynthetic capacity and environmental adaptation [8]. Likewise, the stem characteristics such as the bole morphology and internode length are applied to differentiate between the tall and dwarf varieties [7,9]. Similarly, these macro-morphological features are extremely “plastic” in nature, with a high degree of variation depending on the soil fertility, water availability, and the age of plants. Such phenotypic plasticity tends to cause a “taxonomic fog”, in which environmental noise obscures the real genetic identity of cultivars, promoting genetic erosion and the misidentification of germplasm [10,11].

To complement the limitations of macro-morphological characterization, palynology, the study of pollen morphology can provide additional diagnostic information for plant characterization. Pollen grains contain the male gametophytic material and possess morphological features such as size, shape, and exine ornamentation that can provide useful taxonomic and comparative characters and are generally less influenced by environmental conditions than many vegetative traits [12,13]. Similarly, pollen traits will exhibit lower intra-varietal variation compared to vegetative and nut traits, proving to be a more reliable marker for cultivar differentiation [14]. Moreover, conventional light microscopy may provide limited resolution for detecting subtle differences in pollen morphology and exine surface characteristics among closely related coconut varieties. To date, the relationships between pollen morphological traits, including the polar-to-equatorial (PW/PL) ratio, and macroscopic fruit or yield-related traits in coconuts remain insufficiently explored [15,16]. Therefore, whether variation in pollen morphology is associated with broader vegetative, reproductive, or fruit characteristics requires further investigation. The novelty of the present study lies in its integrated multidimensional morphometric approach, combining conventional plant and fruit morphological traits with pollen morphometric and ultrastructural characteristics across ten farmer-grown aromatic coconut varieties. By integrating observations across different biological scales, the study provides a broader characterization of phenotypic variation among the evaluated varieties. High-resolution scanning electron microscopy further enabled detailed characterization of pollen exine surface architecture, including verrucate, rugulate, and foveolate ornamentation patterns observed among the evaluated varieties.

The combined assessment provides complementary morphological and palynological evidence for the characterization and authentication of aromatic coconut varieties. Specifically, the study aims: (1) to quantify phenotypic variability through vegetative (leaf and stem) and quantitative nut morphometrics; (2) to characterize pollen morphology by measuring polar length and equatorial width and calculating the pollen size index to assess variation in pollen dimensions among coconut varieties; and (3) to characterize pollen exine ultrastructure using scanning electron microscopy and assess variation in surface ornamentation among the evaluated coconut varieties.

## 2. Results

### 2.1. Phenotypic and Palynological Variation in Coconut Varieties

The analysis of variance revealed significant differences among the ten coconut varieties for several phenotypic traits (Table 1). Highly significant varietal effects (*p* < 0.001) were observed for plant height (PH), stem girth (SG), inflorescence length (IL), number of fruits per bunch (NFB), total midrib length (TML), petiole length (PLL), spathe width (SW), fruit weight (FW), and fruit hole spacing (FHS). Significant varietal effects were also detected for size index (SI), number of female flowers (NFF), leaflet length (LFL), leaflet scar spacing (LLS), and leaf scar spacing (LSS). Prior to the analysis, the assumptions of normality and homogeneity of variances were assessed using the Shapiro–Wilk and Levene’s tests, respectively (Supplementary Table S1). Most traits satisfied both assumptions and were analyzed using ANOVA followed by Tukey’s HSD test, whereas PW/PL and TML showed significant departures from normality and were evaluated using an appropriate alternative analytical approach. In contrast, no significant varietal differences were detected for pollen length (PL), pollen width (PW), pollen width-to-length ratio (PW/PL), number of bunches per palm (NBP), leaflet width (LLW), number of leaflets (NLL), or total soluble solids (TSS) (Table 1). For pollen width (PW), the post hoc comparison likewise placed all varieties within the same statistical group, indicating no significant pairwise differences among varietal means.

**Figure 1:**
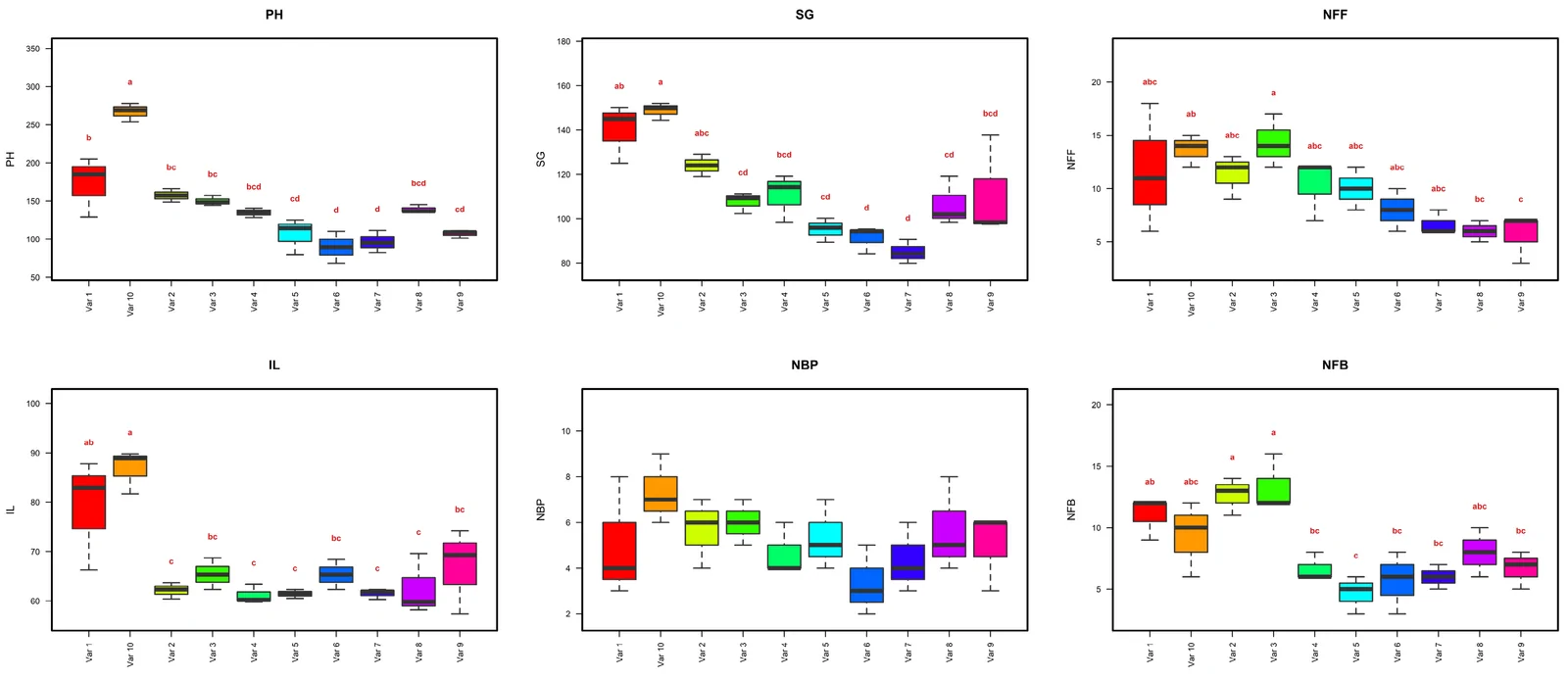

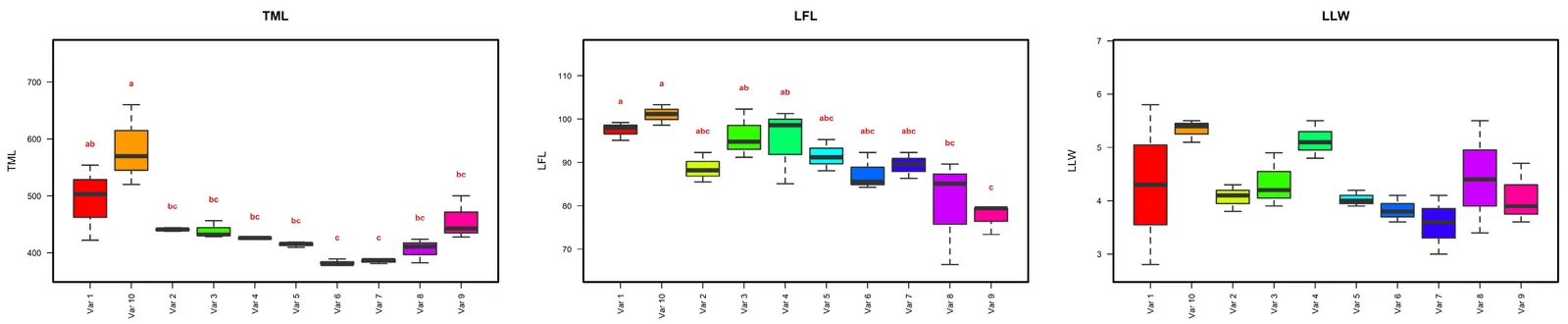
Boxplots showing variation in morpho-agronomic traits among the coconut varieties. The box represents the interquartile range (Q1–Q3), with the lower and upper boundaries corresponding to the first and third quartiles, respectively, and the horizontal line within each box indicating the median. Whiskers extend to the minimum and maximum values observed within the non-outlier range. Different letters above the boxes indicate statistically significant differences among varieties (*p* < 0.05) based on Tukey’s HSD test for traits analyzed using ANOVA. Normality and homogeneity of variances were assessed using the Shapiro–Wilk and Levene’s tests, respectively, before parametric analysis (Supplementary Table S1).

**Figure 2:**
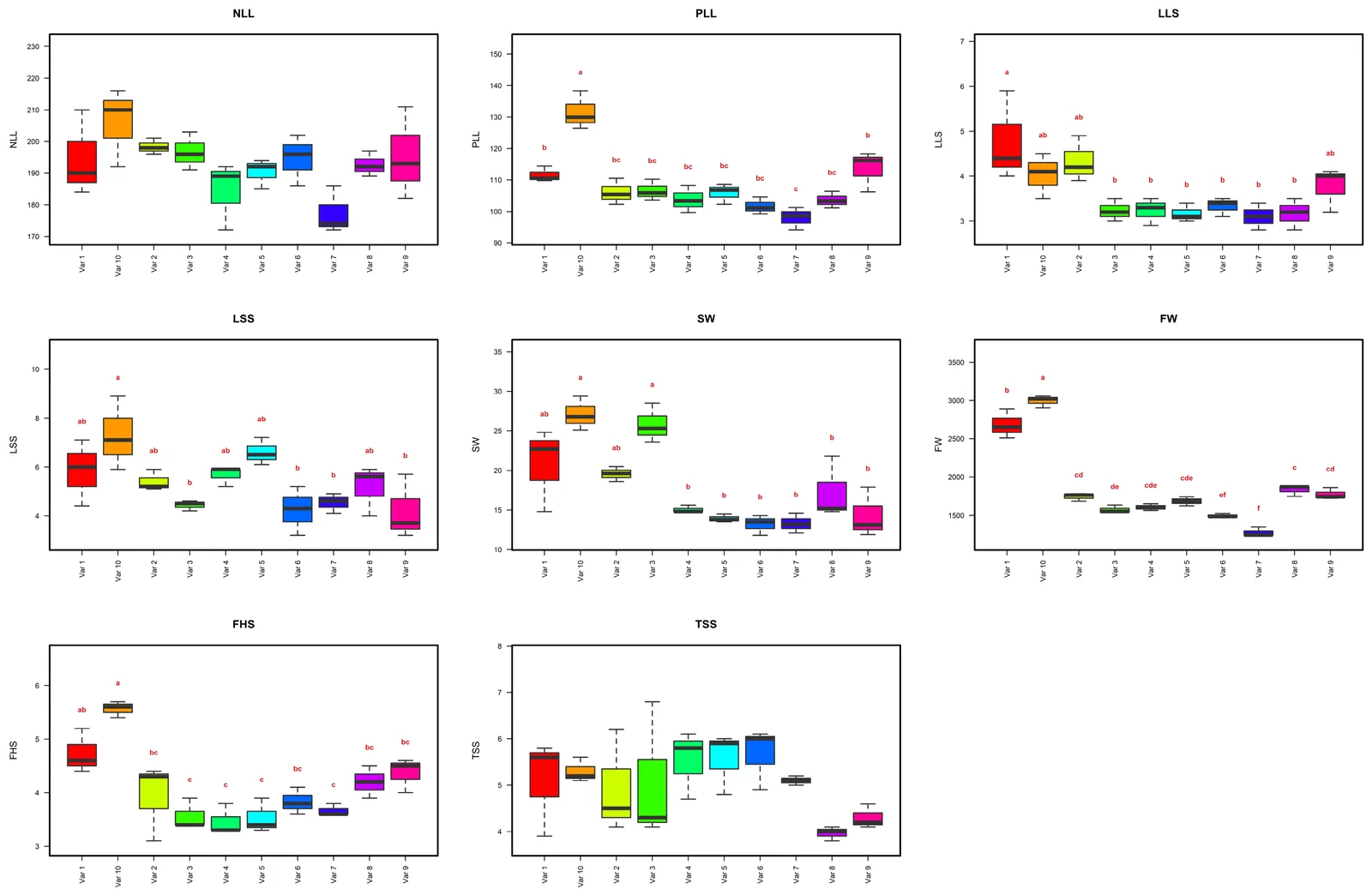
Boxplots showing variation in yield and fruit quality traits among the coconut varieties. The box represents the interquartile range (Q1–Q3), with the lower and upper boundaries corresponding to the first and third quartiles, respectively, and the horizontal line within each box indicating the median. Whiskers extend to the minimum and maximum values observed within the non-outlier range. Different letters above the boxes indicate statistically significant differences among varieties (*p* < 0.05) based on Tukey’s HSD test for traits analyzed using ANOVA. Normality and homogeneity of variances were assessed using the Shapiro–Wilk and Levene’s tests, respectively, before parametric analysis (Supplementary Table S1).

The mean values and variability of the evaluated traits are presented in Supplementary Table S2. Among the significant phenotypic traits, plant height ranged from 89.33 ± 20.15 in Var 6 to 267.00 ± 1.52 in Var 10. According to the mean comparison results, Var 10 differed significantly from the lowest height group represented by Var 6, whereas several intermediate varieties showed overlapping statistical groupings (Figure 1). Stem girth also differed significantly among varieties, with mean values ranging from 84.90 ± 5.32 in Var 7 to 148.73 ± 3.12 in Var 10. The post hoc comparison test showed that Var 10 belonged to the upper statistical group, whereas Var 6 and Var 7 were included in the lower group (Figure 1 and Figure 2). Significant differences were observed for reproductive and inflorescence-related traits. The mean number of female flowers ranged from 5.67 ± 2.31 in Var 9 to 14.33 ± 2.52 in Var 3, with Var 3 differing from the lowest statistical group represented by Var 8 and Var 9. Inflorescence length ranged from 61.13 ± 1.98 in Var 4 to 86.80 ± 4.45 in Var 10, with Var 10 belonging to the highest statistical group. The number of fruits per bunch ranged from 4.67 ± 1.53 in Var 5 to 13.33 ± 2.31 in Var 3, with significant separation between the highest and lowest statistical groups. Total midrib length differed significantly among varieties, ranging from 382.90 ± 5.12 in Var 6 to 583.40 ± 7.21 in Var 10. The post hoc comparison placed Var 10 in the upper statistical group and Var 6 and Var 7 in the lower group (Figure 1). Leaflet length ranged from 77.40 ± 3.42 in Var 9 to 101.03 ± 2.14 in Var 10, with Var 10 and Var 1 belonging to the upper statistical group, whereas Var 9 was assigned to the lowest group. Petiole length also varied significantly among varieties, ranging from 98.00 ± 3.61 in Var 7 to 131.53 ± 6.21 in Var 10. Var 10 formed a distinct upper statistical group, while Var 7 was included in the lower group. Significant differences were also observed for leaflet scar spacing and leaf scar spacing, with several varieties showing overlapping statistical groupings.

**Figure 3:**
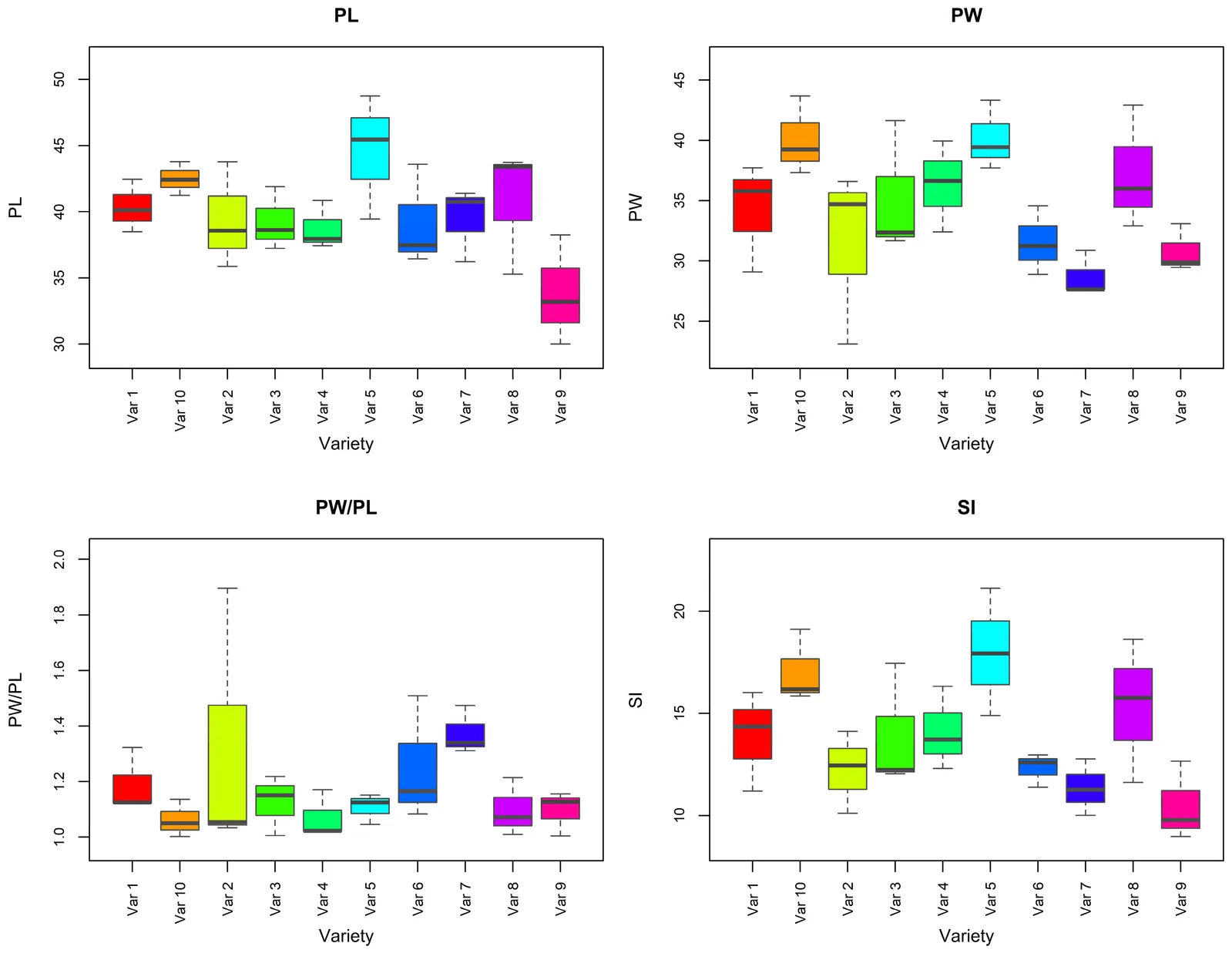
Boxplots showing variation in pollen traits among the coconut varieties. The box represents the interquartile range (Q1–Q3), with the lower and upper boundaries corresponding to the first and third quartiles, respectively, and the horizontal line within each box indicating the median. Whiskers extend to the minimum and maximum values observed within the non-outlier range.

Among the fruit-related traits, fruit weight showed the strongest varietal differentiation, ranging from 1275.00 ± 62.30 g in Var 7 to 2994.00 ± 165.40 g in Var 10 (Figure 2). Post hoc comparison test separated Var 10 and Var 1 from the lower fruit-weight groups, whereas several intermediate varieties showed overlapping statistical groups. Fruit hole spacing also differed significantly among varieties, ranging from 3.47 ± 0.29 in Var 4 to 5.57 ± 0.15 in Var 10. The distribution of phenotypic, fruit-related, and palynological traits among varieties is illustrated in Figure 1, Figure 2 and Figure 3. The boxplots were used to visualize the distribution, median, and variability of individual trait measurements, whereas statistical differences among varieties were determined using the corresponding inferential statistical procedures described in the statistical analysis section.

### 2.2. Genetic Variability Across Morphological and Yield Traits

The analysis of genetic parameters (Table 2) across the ten coconut varieties revealed considerable variability and high potential for improvement in several key morpho-agronomic traits. Plant height (PH) exhibited a wide range (68.4–278 cm) with a mean of 141.99 cm, high genotypic coefficient of variation (GCV = 32.61%), and phenotypic coefficient of variation (PCV = 34.9%), accompanied by very high broad-sense heritability (H_b_^2^ = 0.87) and substantial genetic advance as percentage of mean (GAM = 62.79%), indicating strong additive genetic control and excellent prospects for direct phenotypic selection to enhance stature-related vigor. Fruit weight (FW) showed the highest absolute genetic advance (GA = 989.4) and exceptionally high heritability (H_b_^2^ = 0.97), with GCV (26.14%) nearly equaling PCV (26.55%) and GAM at 53.03%, underscoring its predominantly genetic determination and high response to selection for yield improvement in aromatic coconuts. Similarly, number of female flowers per inflorescence/branch (NFB) displayed high GCV (30.55%), PCV (37.19%), heritability (0.67), and GAM (51.69%), suggesting effective gains possible through breeding for enhanced reproductive potential. Spathe width (SW) and number of bunches per palm (NBP) or related bunch traits also showed moderate to high variability (GCV 24.09% for SW; GAM 41.24% for SW), with good heritability (0.69 for SW) and moderate genetic advance, pointing to selectable components of nut quality and productivity.

Traits linked to vegetative architecture, such as stem girth (SG), internode length (IL), petiole length (PLL), and total mid rib length (TML), generally exhibited moderate heritability (0.64–0.79) and GAM values (13–28%), with lower environmental influence (ECV often < 10%), making them agreeable to moderate selection gains. The predominance of high heritability coupled with moderate-to-high GAM in economically important traits like FW, PH, NFB, and SW highlights the coconut varieties as a promising genetic resource for targeted breeding aimed at improving yield, nut size, and plant vigor.

### 2.3. Correlation Analysis of Morphological Yield Characteristics

**Figure 4:**
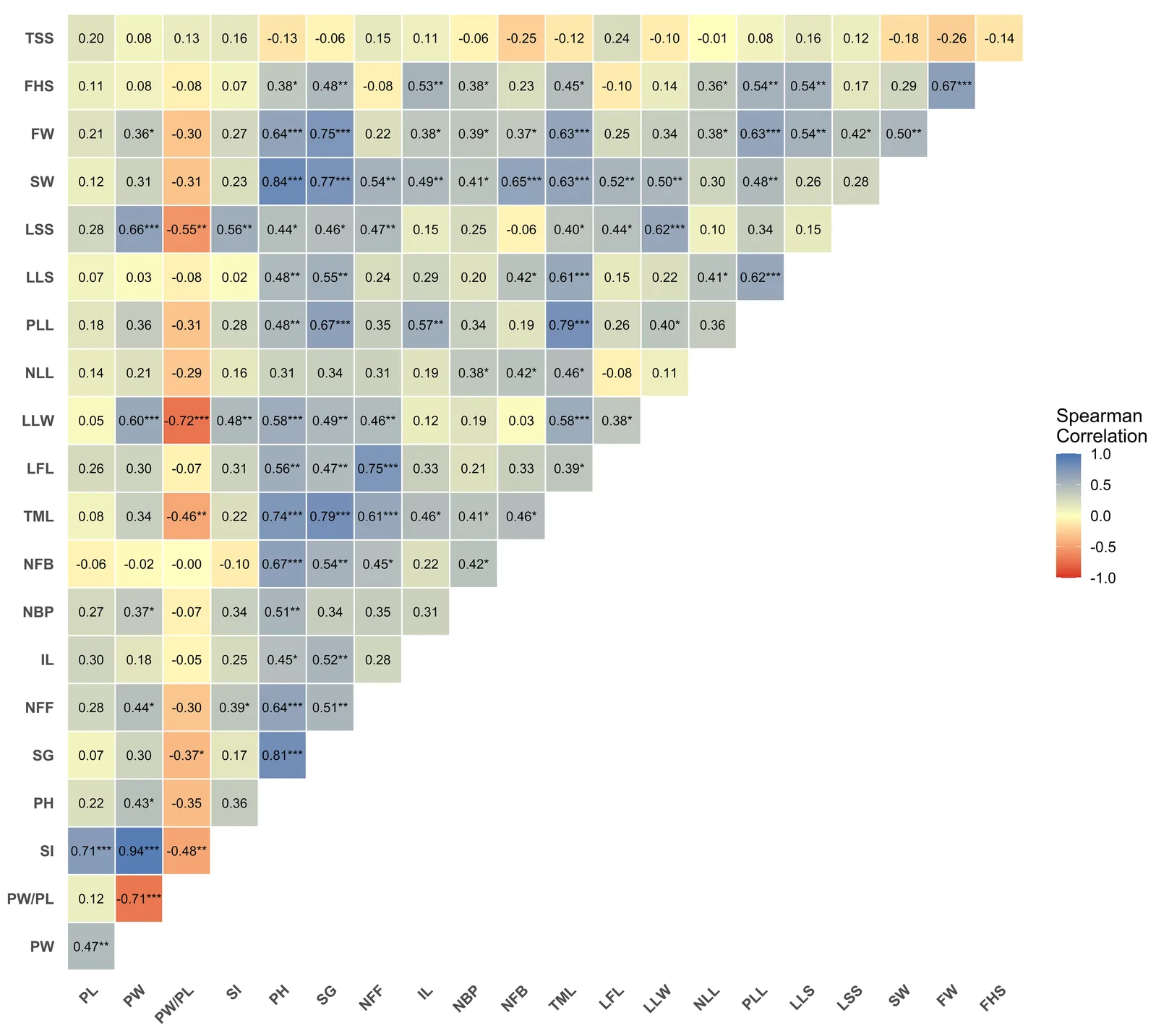
Spearman correlation matrix among 21 morpho-agronomic traits evaluated across ten coconut varieties. Blue shades indicate positive correlations, while red/orange shades indicate negative correlations. Asterisks denote statistical significance: * *p* < 0.05, ** *p* < 0.01, and *** *p* < 0.001.

The correlation matrix analysis among the 23 morpho-agronomic traits in the ten aromatic coconut varieties revealed a complex network of inter-trait relationships, dominated by strong positive associations within vegetative and stature-related characters while showing more limited or variable linkages with reproductive and nut metrics (Figure 4). Plant height (PH) exhibited highly significant positive correlations with nearly all vegetative traits, including stem girth (SG: r = 0.81 ***), total midrib length metric (TML: r = 0.74 ***), leaflet length (LFL: r = 0.60 ***), petiole length (PLL: r = 0.77 ***), and several leaf/stem spacing measures (LLS, LSS, NLL; r values 0.48–0.84 ***), indicating a coordinated genetic architecture underlying overall plant vigor and architecture in these aromatic varieties. Similarly, SG and TML formed a tight cluster of strong intercorrelations (r often > 0.70 ***), reflecting integrated development of stem thickness, leaf production, and height components. Fruit weight (FW) displayed significant positive associations with SW (r = 0.82 ***), PH (r ≈ 0.71 ***), SG (r ≈ 0.61 ***), TML (r ≈ 0.83 ***), and several vegetative traits (r = 0.57–0.79 ***), suggesting that larger, more vigorous palms tend to support heavier nuts and seeds, with potential implications for yield improvement through selection for stature. Reproductive traits such as number of female flowers per inflorescence/branch (NFB) and bunches per palm (NBP) showed moderate positive links to vegetative vigor (NFB with PH, SG, TML; r = 0.41–0.72 ***), but weaker or non-significant ties to nut metrics, highlighting partial independence between floral/fruit-set potential and final nut size/weight. In contrast, pollen traits showed distinct associations among grain dimensions, including a strong negative correlation between pollen width/length ratio (PW/PL) and pollen width (PW; r = −0.71 ***), and a moderate negative association between shape index (SI) and PW/PL (r = −0.48). Traits with consistently low or non-significant correlations across most variables included TSS (total soluble solids), FHS (fruit hole spacing), and certain leaf proportions (LLW).

### 2.4. Pollen Morphometric Characteristics, Morphology, and Exine Ornamentation of Ten Coconut Varieties

**Figure 5:**
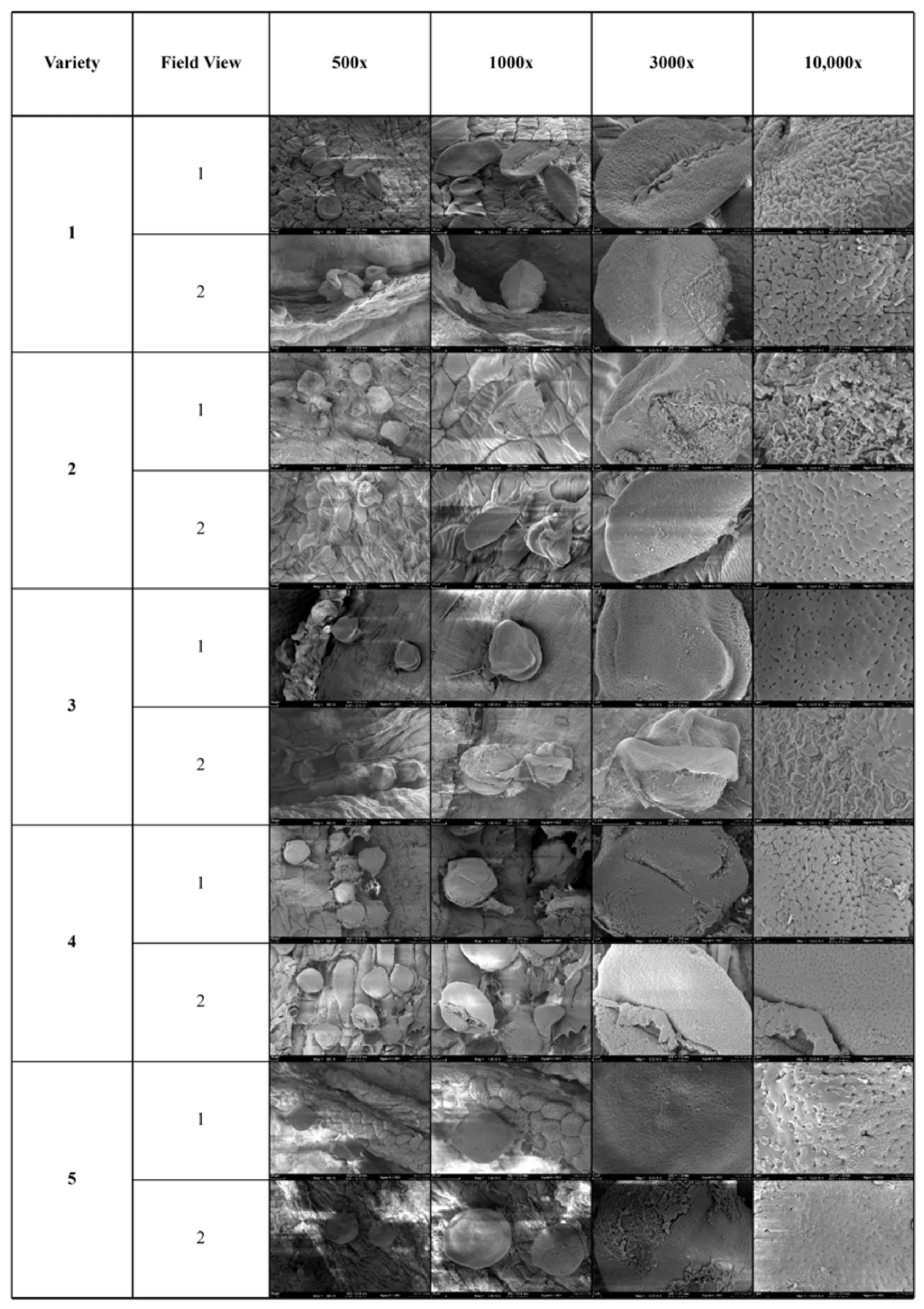
Scanning electron micrographs showing pollen morphology and exine surface characteristics of five coconut varieties (Varieties 1–5). For each variety, two representative SEM views from the same pollen preparation are presented at four magnifications: 500×, 1000×, 3000×, and 10,000×. The 500× views provide an overall view of the pollen grains and their apparent morphology, while the 1000× views provide enlarged views of individual grains. The 3000× views show detailed features of the sulcus/aperture region and pollen surface, while the 10,000× views reveal fine exine ornamentation. Differences in apparent pollen outline among grains within the same preparation represent within-sample morphological variation and differences in grain orientation and SEM field of view.

**Figure 6:**
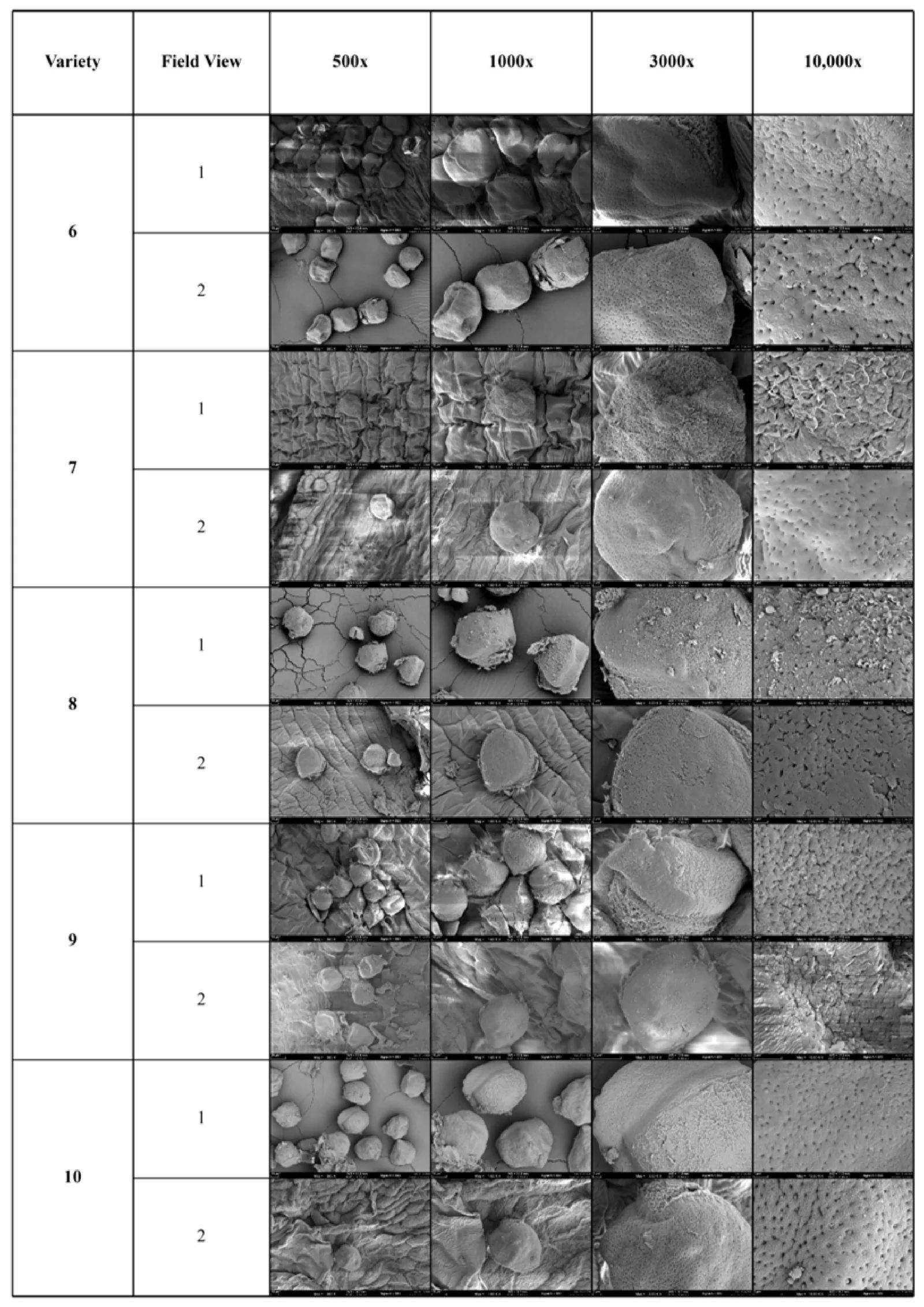
Scanning electron micrographs showing pollen morphology and exine surface features of five coconut varieties (Varieties 6–10). For each variety, two representative SEM views from the same pollen preparation are presented at four magnifications: 500×, 1000×, 3000×, and 10,000×. The 500× views provide an overall view of the pollen grains and their apparent morphology, while the 1000× views provide enlarged views of individual grains. The 3000× views show detailed features of the sulcus/aperture region and pollen surface, while the 10,000× views reveal fine exine ornamentation. Differences in apparent pollen outline among grains within the same preparation represent within-sample morphological variation and differences in grain orientation and SEM field of view.

Pollen morphometric characteristics and surface features of the ten coconut varieties are presented in Table 3 and Table 4, respectively. Pollen grains of all varieties exhibited a monosulcate aperture with a single distal sulcus. The polar length (PL) ranged from 33.82 µm in Var 9 to 44.55 µm in Var 5, whereas pollen width (PW) ranged from 28.71 µm in Var 7 to 40.15 µm in Var 5 (Table 3). The mean PL and PW across all varieties were 39.80 and 34.58 µm, respectively. The PL/PW ratio ranged from 1.06 in Var 10 to 1.37 in Var 7, with an overall mean of 1.17. The size index ranged from 10.42 in Var 9 to 17.89 in Var 5, while pore density ranged from 1.35 pores µm^−2^ in Var 10 to 2.88 pores µm^−2^ in Var 9 (Table 3). Different pollen shapes were observed within the examined samples, including navicular, reniform, ovoid, spherical, and subglobose forms (Table 4; Figure 5 and Figure 6). In most varieties, navicular or boat-shaped pollen grains were commonly observed, together with spherical, ovoid, or subglobose forms. Because pollen shape may be affected by sample preparation and dehydration during SEM processing, these observations are presented descriptively without further interpretation.

SEM observations showed variation in exine surface patterns among the varieties (Table 4; Figure 5 and Figure 6). The observed ornamentation included tectate-rugulate, foveolate-granulate, finely reticulate-perforate, punctate-foveolate, and rugulate-foveolate patterns. Var 1 exhibited tectate-rugulate to foveolate-granulate ornamentation, whereas Var 2 and Var 4 showed finely reticulate-perforate surfaces. Var 3 displayed a tectate-rugulate-foveolate pattern with granular elements, while Var 5 showed tectate-rugulate-granulate ornamentation with subtle gemmate projections (Figure 5). Var 6 and Var 10 exhibited punctate-foveolate ornamentation with elevated ridges and micro-pores. Var 7 showed tectate-rugulate-foveolate-granulate ornamentation, whereas Var 8 and Var 9 exhibited finely reticulate-perforate patterns (Table 4 & Figure 6). The SEM micrographs presented in Figure 5 and Figure 6, illustrate the pollen aperture, overall shape, and exine surface features observed among the ten varieties.

### 2.5. Principal Component and Cluster Analysis of Morphological and Pollen Traits

**Figure 7:**
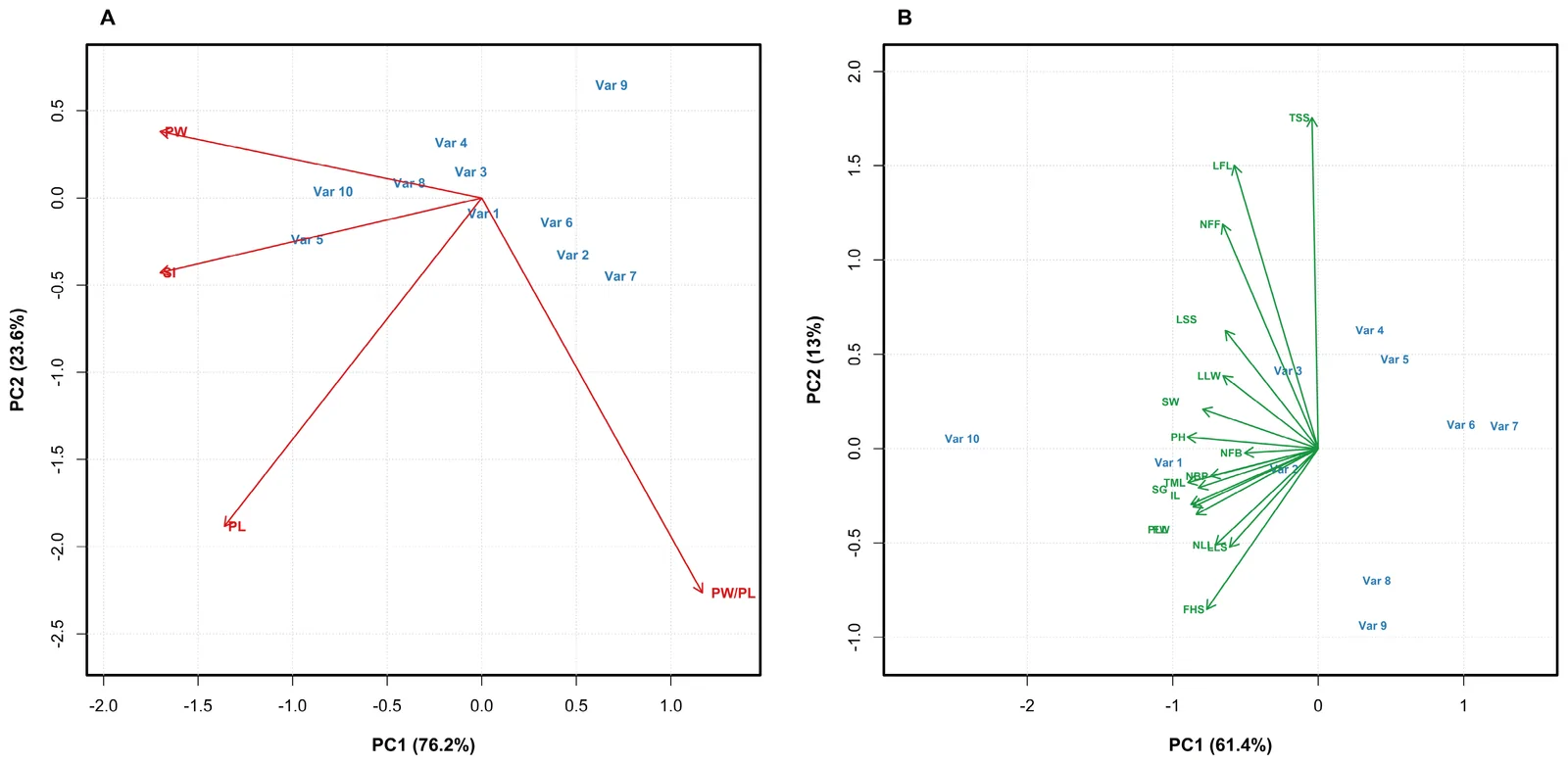
Principal component analysis biplots illustrate the multivariate relationships among coconut varieties based on (**A**) pollen traits and (**B**) morpho-agronomic traits. Vectors represent trait loadings, and text labels represent individual varieties (Var 1 to Var 10). Percentages on axes indicate the proportion of total variance explained by each principal component.

Principal component analysis (PCA) revealed clear patterns of variation among the ten coconut varieties based on both morphological and pollen traits. The PCA of pollen morphometric traits showed that the first two principal components explained 99.8% of the total variation, with PC1 accounting for 76.2% and PC2 accounting for 23.6% (Figure 7A). Pollen width (PW), size index (SI), and pollen length (PL) were the major traits contributing to the separation of varieties. The two varieties (Var 9 and Var 7) differed from the other varieties in their spatial separation, corresponding to their different sizes and shapes of pollen and shape-associated trait configurations.

The PCA based on morphological traits further demonstrated substantial phenotypic diversity among the ten varieties (Figure 7B). The first two principal components explained 74.4% of the total variation, with PC1 accounting for 61.4% and PC2 accounting for 13.0%. The vegetative growth traits plant height (PH), stem girth (SG), total midrib length (TML) and inflorescence length (IL) exhibited a similar loading pattern, suggesting they were closely coupled with the other traits in the overall morphological variation. These traits were differentiated from fruit and yield-related characteristics along the principal component axes. Var 10, Var 4, and Var 5 were distinctly separated from the remaining varieties, reflecting differences in their overall morphological, stature, and yield-related characteristics.

**Figure 8:**
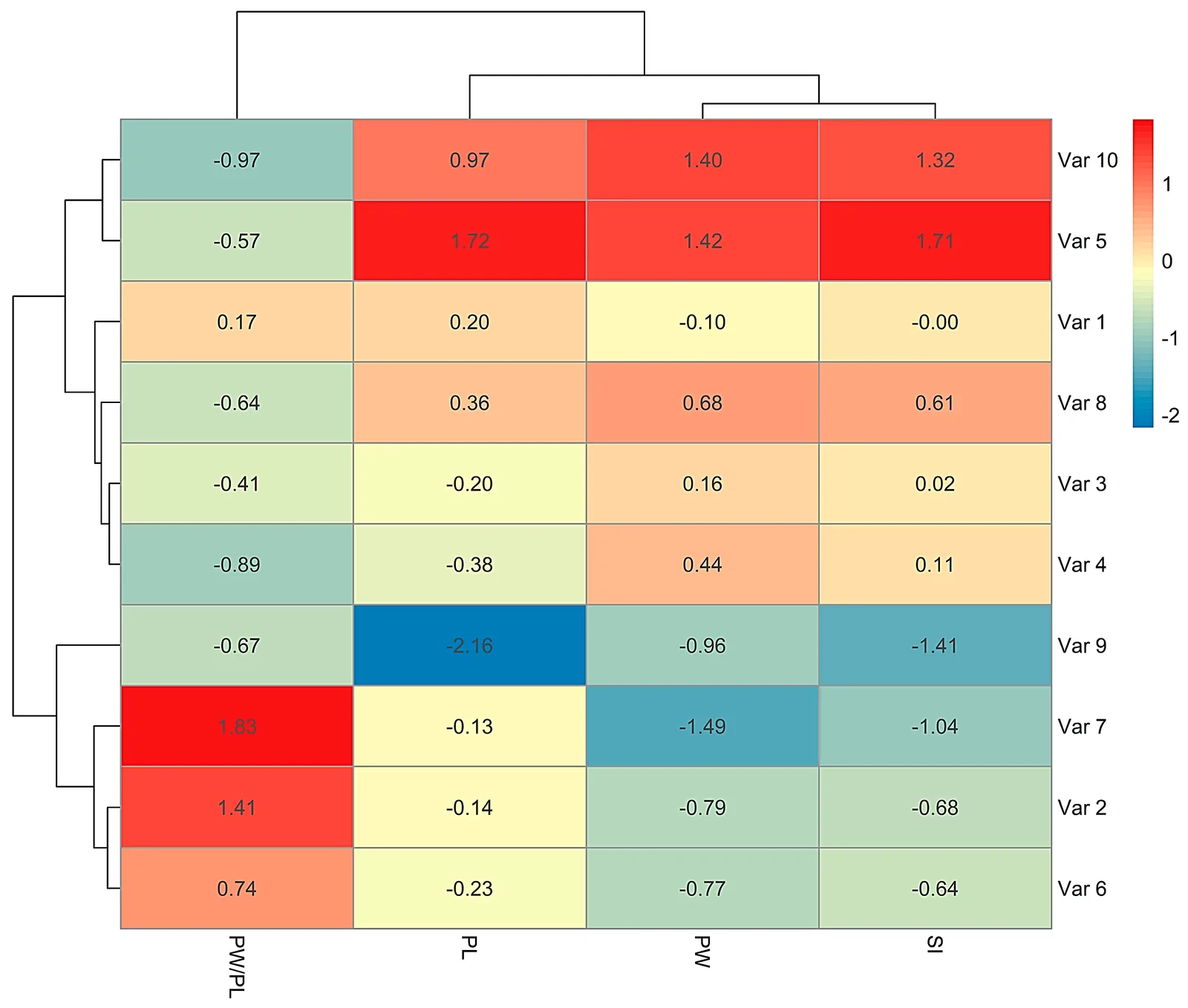
Two-way hierarchical cluster heatmap showing standardized expression levels (Z-scores) of pollen traits (PW/PL, PL, PW, and SI) across the 10 coconut varieties. Euclidean distance clustering and complete linkage group both genotypes (rows) and traits (columns), with color intensity reflecting relative trait magnitude from low (blue) to high (red).

Hierarchical clustering based on standardized pollen dimensions and ratios further separated the ten varieties into distinct groups (Figure 8). The heatmap revealed clear differences in PL, PW, PW/PL ratio, and SI among the varieties. Var 10 and Var 5 formed a distinct cluster characterized primarily by relatively high standardized values for PW and SI. In contrast, Var 7, Var 2, and Var 6 were grouped based on their comparatively higher values for the pollen width-to-length ratio and PL-related variation. Var 9 was distinguished by a strong negative standardized deviation for pollen length, consistent with its relatively smaller pollen dimensions.

## 3. Discussion

The present study demonstrated significant phenotypic diversity among the coconut varieties, particularly for vegetative architecture, reproductive traits, and fruit-related characteristics. The pollen observations obtained using SEM provided additional descriptive information on pollen dimensions, aperture appearance, and exine surface characteristics under the preparation and imaging conditions used in this study. These findings complement previous coconut germplasm studies showing that morphological and reproductive characters can provide useful information for describing variation among coconut populations and accessions [15,16,17,18].

Highly significant genotypic differences (*p* < 0.001) for plant height, stem girth, total midrib length, fruit weight, petiole length, spathe width, and hole spacing, indicating substantial variation among the evaluated varieties for these traits. Sudha et al. [16] similarly recorded large genotypic mean squares for fruit weight and stature traits across eight accessions, while Sivakumar et al. [19,20] observed highly significant variation for palm height, nut yield, and whole-nut weight in dwarf × tall hybrids [19,20]. Subramanian et al. [21,22] and Natarajan et al. [21,22] noted high variability for nut yield, de-husked nut weight, and copra weight in 28 genotypes, and found significant differences for palm height, girth, leaf length, and nut yield among 18 tall germplasms. The genetic variability analysis further indicated that several economically important traits showed relatively high heritability and genetic advance. Fruit weight exhibited high broad-sense heritability and genetic advance, suggesting that the observed variation in this trait was strongly associated with differences among the evaluated varieties under the conditions of the present study. Plant height and reproductive traits, including the number of female flowers, also showed considerable variability and potential usefulness in selection programmes [7,21,22]. The present morphological findings are also consistent with our recent on-farm survey of aromatic coconut populations, which demonstrated substantial variation among populations and provinces under farmer-managed production environments. While the previous study evaluated population–province variation across different on-farm conditions, the present study focuses on variation among ten selected Nam Hom coconut varieties through detailed morpho-agronomic evaluation and additional pollen observations [23].

In contrast, non-significant genotypic variation for pollen length, width, shape ratio, number of bunches per plant, leaflet ratio, number of leaflets and total soluble solids indicate uniformity within this narrow aromatic dwarf-like background. Pollen-size stability falls sharply against broad ranges (55–71 μm length) as observed by Sudha et al. [16] in geographically distant tall and dwarf accessions but matches low variability reported for pollen traits in regional collections [16]. PW/PL ratio of 1.06 to 1.37 is still within the subprolate to prolate-spheroidal ranges that have been documented globally thus reinforcing that shape is genetically stable and environmentally insensitive, a major advantage for authentication [13,14].

SEM observations also revealed differences in the appearance of exine surface features among the examined varieties. The observed descriptions included tectate-rugulate, foveolate-granulate, finely reticulate-perforate, punctate-foveolate, and related surface patterns. These characteristics were recorded descriptively in Table 4 and illustrated in the SEM micrographs (Figure 5 and Figure 6). The differences in surface appearance may provide useful descriptive characters for comparing the pollen samples examined in this study; however, they should be regarded as candidate descriptors under the present sampling and SEM preparation conditions rather than definitive variety-specific diagnostic markers. The exine descriptions generally fall within the range of surface patterns previously reported for coconut pollen. Sudha et al. [16] documented variation in pollen surface ornamentation among coconut accessions, including granulate, reticulate, and foveolate patterns.

Correlation analysis revealed clear relationships among several vegetative, reproductive, and yield-related traits in the ten coconut varieties. Plant height showed strong positive associations with stem girth, total midrib length, leaflet length, and petiole length, indicating coordinated variation among traits related to overall palm architecture. Similarly, the positive associations among stem girth, total midrib length, and other vegetative characteristics suggest that larger palms tended to possess more developed vegetative structures. This pattern indicates coordinated phenotypic variation in plant architecture within the evaluated varieties rather than independent variation of individual vegetative traits.

Fruit weight was also positively associated with several vegetative traits, including plant height, stem girth, and total midrib length. The strong association between fruit weight and total midrib length is particularly notable because it suggests that palms with greater vegetative development also tended to produce heavier fruits within the present population. However, these relationships should be interpreted as phenotypic associations and do not necessarily indicate direct causal relationships between vegetative growth and fruit weight. Similar positive relationships between palm growth, leaf characteristics, and yield-related traits have been reported in previous coconut germplasm studies by Sivakumar et al. [19,20]; Subramanian et al. [21,22]; and Natarajan et al. [21,22].

The correlation analysis also showed that reproductive traits were associated with vegetative characteristics to varying degrees. The number of female flowers and fruit-related traits showed weaker and more variable relationships with some morphological characters than the stronger correlations observed among vegetative traits. Similar relationships between vegetative and reproductive characteristics have been reported in coconut germplasm, where traits such as stem girth and inflorescence characteristics showed significant associations, while fruit and quality traits exhibited distinct correlation patterns [24,25]. This indicates that reproductive variation among the evaluated varieties was not completely accounted for by differences in plant size or vegetative vigor. The partial independence among trait groups is also consistent with previous multivariate analyses of coconut germplasm, which have shown that vegetative, reproductive, fruit, and quality traits can exhibit distinct patterns of association [23,24].

The morphological PCA provided a multivariate summary of variation among the evaluated coconut varieties based on the quantitative traits included in this study. The separation of varieties along the principal component axes was associated with differences in plant stature, vegetative development, reproductive characteristics, and fruit-related traits, consistent with trait patterns reported in previous multivariate studies of coconut germplasm [10,11,21,22]. The first two principal components explained 74.4% of the total morphological variation, with PC1 accounting for 61.4% and PC2 accounting for 13.0%. Plant height, stem girth, total midrib length, and inflorescence length showed similar loading patterns, indicating that these traits contributed jointly to the major axis of morphological variation. The similar loading directions of these vegetative traits are consistent with their positive correlations observed in the correlation analysis, indicating that coordinated variation in plant architecture was an important source of diversity among the evaluated varieties. Previous studies of coconut germplasm have likewise shown that combinations of vegetative, reproductive, fruit, and quality traits can account for substantial portions of the observed multivariate variation [23,24]. Fruit and yield-related characteristics were distributed differently along the principal component axes, demonstrating that the morphological variation among varieties was not determined solely by palm size. Var 10, Var 4, and Var 5 were distinctly separated from several of the remaining varieties, reflecting differences in their combinations of vegetative, reproductive, and yield-related characteristics. This separation provides a multivariate representation of the phenotypic differences observed across individual traits but should not be interpreted as evidence of fixed biological groups or superior varietal types.

For the pollen morphometric dataset, PC1 and PC2 together explained 99.8% of the total variation, indicating that most of the variation in the measured pollen dimensions and ratios was captured by these two components. Pollen width, size index, and pollen length were major contributors to the separation of the sampled varieties. Var 9 and Var 7 occupied distinct positions in the ordination, reflecting differences in their pollen dimensional measurements and ratios. The high proportion of variation explained by the first two components indicates that the measured pollen variables were strongly summarized by these principal axes within the present dataset. Similar multivariate approaches have been applied to pollen datasets to summarize quantitative morphological variation and identify patterns of similarity among samples [15].

Hierarchical clustering based on standardized pollen dimensions and ratios further grouped the varieties according to similarities in PL, PW, PW/PL ratio, and size index. Var 10 and Var 5 formed a group associated with relatively higher standardized PW and SI values, whereas Var 7, Var 2, and Var 6 showed different combinations of pollen dimensions and ratios. Var 9 was distinguished by relatively lower pollen length values. The use of quantitative pollen characteristics for multivariate differentiation is also consistent with previous work in coconut, where hierarchical clustering based on pollen characteristics separated 20 coconut varieties into major groups [15].

From a breeding standpoint, the high heritability and GAM for fruit weight, plant height, and female flowers (53–63%) indicate that selecting top performers from Cluster 1 (especially Var 1 and Var 5) may useful candidates for further evaluation and selection, which is broadly consistent with findings from Indian tall coconut improvement programmes [21,22]. Pollen stability ensures consistent reproductive biology, reducing hybridization failures common in coconut due to long generation time and complex flowering [4]. The variety-specific exine patterns enable a simple SEM-based identification key, fulfilling the authentication benchmark and extending the palynotaxonomic utility shown by Halbritter et al. [14]. Compared with broader literature, the Nam Hom collection exhibits lower pollen-size variability than Sudha et al. [16] global panel but higher exine micro-diversity within a narrow aromatic background, suggesting that within-group selection refines surface sculpture, an observation with direct implications for virgin coconut oil and tender-coconut markets where consistent pollen viability and nut quality are paramount.

The integrated analysis of morpho-agronomic and pollen-related variables also helped visualize different levels of variation within the evaluated material. The morphological PCA demonstrated substantial diversity in vegetative, reproductive, and fruit-related traits, whereas pollen PCA and hierarchical clustering summarized differences in measured pollen dimensions and ratios. Rather than demonstrating a validated authentication system, these complementary analyses provide an exploratory framework for understanding how macro-morphological and pollen-related characteristics vary within the present collection.

This study has several limitations. The evaluation was restricted to ten selected coconut varieties and was conducted under the specific environmental conditions of the study area; therefore, the findings may not represent the full diversity of aromatic coconut varieties. The pollen observations were based on the sampled material and specific SEM preparation and imaging conditions, and the observed exine patterns should therefore be considered descriptive rather than definitive diagnostic markers. In addition, the study did not include molecular markers, pollen viability or fertility assessments, controlled hybridization experiments, or multi-location evaluations. Future studies integrating molecular, reproductive, and multi-environment approaches would provide further validation of the observed morphological and pollen-related variation.

## 4. Materials and Methods

### 4.1. Study Location and Climate Conditions

Field experiments were conducted on farmer-managed coconut plots in Trang province, southern Thailand (7°38′06.5″ N 99°28′25.9″ E). Trang province is located within the tropical monsoon climatic zone of the Thai Peninsula and is characterized by generally warm and humid conditions with a distinct rainy season and a comparatively drier period. The regional climate is influenced by the southwest and northeast monsoons, resulting in substantial seasonal rainfall. The study was conducted under the prevailing environmental conditions of Trang province. During the study period, monthly temperature and rainfall conditions reflected the typical warm and humid tropical environment of the region. The mean air temperature generally ranged from approximately 26.4 to 27.2 °C, while relative humidity ranged from approximately 73.9 to 90.5%. Wind speeds were generally low, ranging from approximately 2.1 to 5.5 knots. Monthly precipitation during the relatively dry period varied from approximately 4.8 to 106.0 mm. The soils in Trang province are mainly acidic tropical soils, commonly classified within Ultisol and related soil groups, with considerable variation according to local parent material and topography. The soils in the study area were generally well drained and ranged from sandy clay loam to clay in texture, reflecting the typical soil conditions under coconut-growing systems in the region.

### 4.2. Plant Material and Experimental Design

A total of ten farmer-grown aromatic coconut varieties were evaluated in the present study at a single study location. These varieties represent locally cultivated aromatic coconut types that are maintained and grown by farmers and are recognized based on their local identity and phenotypic characteristic and aromatic attributes. The ten varieties were selected based on their local cultivation importance, recognized aromatic characteristics, phenotypic distinctiveness, and relevance to regional coconut production. Detailed information for each variety, including the local name, variety code, palm age, stature class, sampling location is provided in Supplementary Table S3.

For each variety, three healthy and reproductively mature individual palms were selected for sampling. Palms showing visible symptoms of severe pest infestation, disease, or substantial mechanical damage were excluded from the study. The individual palm was defined as the biological experimental unit and represented one independent observation in the statistical analysis. Thus, the sampling structure consisted of 10 varieties × 3 independent palms per variety. Variety was treated as a fixed effect because the objective was to compare the specific farmer-grown coconut varieties included in the study. The three palms within each variety provided biological replication and were used to estimate within-variety variation.

For traits measured repeatedly within a palm, including pollen grains and scanning electronic microscope observations, individual grains were considered subsamples or technical observations rather than independent biological replicates. These observations were summarized at the palm level, where applicable, before statistical analysis to avoid pseudo replication. Therefore, the sampling followed a hierarchical structure in which individual palms were nested within varieties, pollen samples were obtained from each palm, and individual pollen grains and SEM observations represented subsampling units rather than independent biological replicates.

### 4.3. Morpho-Agronomic Trait Assessment

During the peak fruiting season (January–August 2026), a comprehensive set of 17 morpho-agronomic traits was systematically recorded in aromatic coconut palms following IPGRI standardized descriptors adapted for this type, ensuring phenological consistency and minimizing seasonal bias [26,27]. The quantification of vegetative and morphometric characteristics was done with calibrated instruments; plant height (cm) between soil surface and apical meristem by using a measuring pole, average internode distance (cm) between leaf scars, stem girth (cm) at 1.3 m above the ground using a flexible tape, total leaf scars, stem diameter (cm) in lower and upper parts, number of functional and unopened leaves, length of leaves (cm) and length and width of leaflets (cm). There were four reproductive characteristics length and width of the spathe (cm), length of the inflorescence (cm) along the peduncular axis, and number of female flowers per inflorescence. The five fruit and nut characteristics included hole spacing (cm) using digital vernier calipers (with accuracy of 0.01 mm), weight of fruit (kg) using a calibrated electronic balance, and number of nuts per bunch (mature and immature). Each measurement was taken three times on each palm and results were averaged to reduce the error of the observer, within-plant variability and increase the statistical reliability.

### 4.4. Scanning Electron Microscopy of Pollen Grains

For each coconut variety, three independent pollen samples were collected for morphological evaluation. Each sample consisted of ten randomly selected pollen grains; therefore, a total of 30 pollen grains were examined per variety. The ten pollen grains within each sample were treated as subsamples, whereas the three independently collected samples represented the replicate sampling units for each variety. Pollen grains were collected during the early morning period (about 8:00 a.m.–10:00 a.m.), when pollen viability is optimal and transferred immediately to the laboratory for processing [28].

Standard chemical fixation protocols used to prepare the scanning electronic microscopy sample were adapted to the morphology preservation of pollen grains. Pre-fixation was ensured by immersing samples of pollen into 2.5% glutaraldehyde in phosphate buffer (pH 7.2) [28]. Then samples were washed twice successively with 0.1 M sodium phosphate buffer (pH 7.2) for 20 min to eliminate the surplus fixative and buffer salts. Post-fixation was performed using 1% osmium tetroxide (OsO_4_) in distilled water for 1 h, which provides secondary fixation and improves contrast for electron microscopy visualization [16,28]. This was followed by three rinses with distilled water for 30 min each to ensure complete removal of osmium tetroxide. Dehydration was performed using a graded ethanol series to gradually replace water and prepare samples for critical point drying. Samples were serially immersed in 30% ethanol for 10 min, followed by 50, 70, 90, and 100% ethanol solutions, each for 10 min, allowing gradual water displacement and minimizing cellular shrinkage. Critical point drying was performed for 1 h 30 min using a critical point dryer (Quorum, K850, Quorum Technologies, Laughton, East Sussex, UK) to remove all residual organic solvents under supercritical conditions, preserving the three-dimensional ultrastructure of pollen grains without distortion. Dried samples were stored in a desiccator for 24 h prior to coating to ensure complete moisture removal. Specimen mounting and sputter-coating involved placing pollen samples on SEM stubs and applying a 90 s gold coating using thermal evaporation to establish electrical conductivity and prevent accumulation during imaging. Microscopic examination was conducted using a scanning electron microscope (Zeiss; Merlin compact, Carl Zeiss Microscopy GmbH, Oberkochen, Germany) operating at multiple magnifications ranging from 100× to 10,000× to visualize pollen grain morphological features at various scales, including aperture architecture, surface ornamentation, and overall grain morphology [28]. For SEM observation and the acquisition of representative group micrographs, three pollen samples were examined per variety. Multiple pollen grains and SEM fields within the same sample or preparation were considered technical observations and were used to characterize pollen morphology and exine features; they were not treated as independent biological replicates.

Pollen length (PL) and pollen width (PW) were measured from calibrated SEM images using ImageJ 1.54g (running on Java 1.8.0_345 64-bit. Image calibration was performed using the scale bar provided with each SEM micrograph. PL was measured along the longest axis of an individual pollen grain, whereas PW was measured perpendicular to the longest axis at the widest point. Measurements were obtained from thirty grains per variety, and the resulting values were used to calculate the mean and standard deviation for each variety. Pollen pore density was determined via ImageJ calibrated ROI measurements using the formula:

$$\mathrm{Pore}\;\mathrm{density}\;(\mathrm{pores}/\mu m^{2})=\frac{\mathrm{No}.\;\mathrm{of}\;\mathrm{pores}}{\mathrm{ROI}\;\mathrm{area}\;(\mathrm{pixels})\times {\left(\frac{\mu m}{\mathrm{pixel}\;\mathrm{distance}}\right)}^{2}}$$

Additionally, the pollen width-to-length ratio (PW/PL) and pollen size index (SI) were derived from individual measurements of pollen length (PL) and pollen width (PW). The size index was calculated as:

$$SI=\frac{PL\times PW}{100}$$

Pollen shape classes were assigned based on the observed grain outline and the PL-to-PW relationship, using consistent morphological criteria across varieties. Aperture characteristics were classified based on the number, position, and morphology of apertures. Exine ornamentation was classified from SEM micrographs according to the observed surface architecture, including the presence and arrangement of muri, lumina, ridges, foveolae, perforations, granules. Classification was based on repeated observations across the examined pollen grains and SEM fields, and representative micrographs were selected for illustration.

### 4.5. Statistical Analysis

All data analyses were performed using R software (version 4.2.1). Prior to parametric analysis, the assumptions of normality and homogeneity of variance were verified using Shapiro–Wilk and Levene’s tests, respectively. Analysis of variance (ANOVA) was performed to evaluate variation across vegetative, reproductive, and pollen-related traits among the ten coconut varieties evaluated across three replications. Variety was treated as a fixed effect because the objective of the study was to compare the specific coconut varieties included in the experiment, whereas replication was considered a random blocking effect to account for variation among replicates. The statistical model was:

$$Y_{ij}=\mu +G_{i}+R_{j}+\varepsilon_{ij}$$

where $Y_{ij}$ is the observed trait value, μ is the overall population mean, $G_{i}$ is the fixed effect of the i-th variety, $R_{j}$ is the random effect of the j-th replication, and $\varepsilon_{ij}$ is the residual error [29]. Mean comparisons were carried out using Duncan’s multiple range test via the agricolae package (version 1.3-7) at a significance threshold of *p* < 0.05.

Relationships among quantitative morphological and physiological traits were assessed using Spearman rank correlation analysis performed through the *psych* package (version 2.3.6). Spearman correlation was selected to evaluate monotonic relationships among traits while reducing sensitivity to departures from multivariate normality [30]. To handle the combination of continuous metric measurements (such as polar length and width) and qualitative/binary exine descriptors (such as aperture type and shape classes), Factor Analysis of Mixed Data (FAMD) was implemented using the FactoMineR package (version 2.16). PCA and FAMD biplots were constructed based on calculated entry means rather than individual replicate observations to provide a stable representation of multi-trait ideotypes. Furthermore, patterns of trait variation and variety associations were visualized using two-way hierarchical clustering heatmaps [31,32].

## 5. Conclusions

This study demonstrated substantial phenotypic variation among the ten evaluated coconut varieties, particularly in plant height, stem-related traits, reproductive characteristics, and fruit weight. The integrated morpho-agronomic and pollen characterization approach combined macro-morphological measurements with SEM-based observations of pollen dimensions and exine surface characteristics, providing complementary information on variation within the evaluated collection. Multivariate analyses further revealed differences among varieties based on combinations of vegetative, reproductive, fruit-related, and pollen morphometric traits. These patterns provide an exploratory representation of phenotypic variation rather than evidence of fixed biological groups or superior varietal types. The relatively high heritability and GAM values observed for fruit weight, plant height, and number of female flowers suggest that these traits may be useful for identifying promising materials for further evaluation and selection. Pollen dimensions showed relatively limited variation among the evaluated varieties, whereas differences in exine surface appearance provided additional descriptive information for comparing pollen samples. However, the observed pollen and exine characteristics should be regarded as complementary descriptors under the present sampling and SEM conditions rather than definitive markers for varietal authentication.

## Figures and Tables

**Table 1:** Analysis of variance for morpho-agronomic and pollen traits evaluated in ten coconut varieties.

Trait	Replication (df = 2)		Variety (df = 9)			Residuals (df = 18)
	MS	F	MS	F-Value	Sig.	MS
PL	12.484	1.085	22.998	1.999	ns	11.507
PW	13.879	0.752	46.599	2.524	*	18.466
PW/PL	0.008	0.208	0.037	0.959	ns	0.039
SI	7.531	1.417	17.411	3.275	*	5.317
PH	4.674	0.014	8100.972	23.314	***	347.476
SG	171.308	1.644	1261.243	12.100	***	104.232
NFF	1.033	0.129	28.744	3.578	*	8.033
IL	111.809	5.726	226.684	11.609	***	19.526
NBP	2.533	0.884	3.411	1.190	ns	2.867
NFB	11.100	3.808	27.837	9.550	***	2.915
TML	715.990	0.589	10,586.48	8.711	***	1215.26
LFL	28.377	0.838	166.778	4.925	**	33.865
LLW	0.556	1.250	0.905	2.035	ns	0.445
NLL	34.633	0.371	183.219	1.960	ns	93.485
PLL	11.559	0.655	261.340	14.809	***	17.647
LLS	0.014	0.059	1.026	4.187	**	0.245
LSS	0.090	0.094	3.270	3.383	*	0.967
SW	0.651	0.086	79.417	10.489	***	7.572
FW	3957.7	0.538	880,084.9	119.564	***	7360.8
FHS	0.121	0.959	1.308	10.341	***	0.127
TSS	0.506	0.800	0.899	1.422	ns	0.633

Where, ***, **, * indicate significant differences at *p* < 0.001, *p* < 0.01 and *p* < 0.05, respectively; ns indicates non-significant differences. Codes: PH = plant height, SG = stem girth, NFF = number of female flowers per inflorescence, IL = inflorescence length, NBP = number of bunches per palm, NFB = number of fruits per bunch, TML = total midrib length, LFL = leaflet length, LLW = leaflet length-to-width ratio, NLL = number of leaflets, PLL = petiole length, LLS = leaflet scar spacing, LSS =leaf scar spacing, SW = spathe width, FW = fruit weight, FHS = fruit hole spacing (nut), TSS = total soluble solids, PL = pollen length, PW = pollen width, PW/PL = pollen shape ratio, SI = size index.

**Table 2:** Genetic parameters for morpho-agronomic and pollen traits are evaluated in 10 Nam Hom coconut varieties.

Traits	Min	Max	Mean	CD (5%)	GCV (%)	PCV (%)	ECV (%)	H_b_^2^ (BS)	GA	GAM (%)
PH	68.40	278.00	141.99	30.52	32.61	34.90	12.42	0.87	89.15	62.79
SG	79.90	152.00	111.99	18.67	15.61	18.34	9.63	0.72	30.64	27.36
NFF	3.00	18.00	9.77	4.80	24.95	37.78	28.38	0.44	3.31	33.93
IL	57.40	89.80	67.23	7.73	11.15	12.98	6.64	0.74	13.26	19.72
NBP	2.00	9.00	5.23	2.98	4.85	33.28	32.93	0.02	0.08	1.45
NFB	3.00	16.00	8.40	3.08	30.55	37.19	21.21	0.67	4.34	51.69
TML	379.20	660.00	442.90	63.97	11.14	13.92	8.34	0.64	81.3	18.36
LFL	66.40	103.30	90.43	10.62	6.37	9.30	6.79	0.47	8.12	8.98
LLW	2.80	5.80	4.31	1.21	6.87	17.65	16.26	0.15	0.24	5.51
NLL	172.00	216.00	193.03	17.62	2.09	5.67	5.27	0.14	3.05	1.58
PLL	94.10	138.30	108.23	7.30	7.53	8.48	3.90	0.79	14.90	13.76
LLS	2.80	5.90	3.61	0.84	12.98	18.70	13.46	0.48	0.67	18.56
LSS	3.20	8.90	5.34	1.76	14.14	23.76	19.09	0.35	0.93	17.34
SW	11.80	29.40	18.03	5.03	24.09	28.99	16.13	0.69	7.44	41.24
FW	1238	3057	1865.8	149.48	26.14	26.55	4.63	0.97	98.40	53.03
FHS	3.10	5.70	4.09	0.57	14.19	16.34	8.10	0.75	1.04	25.38
TSS	3.80	6.80	5.05	1.31	6.90	16.49	14.98	0.18	0.30	5.95

Where GCV = genotypic coefficient of variation (%); PCV = phenotypic coefficient of variation (%); ECV = environmental coefficient of variation (%). H_b_^2^ = broad-sense heritability; GA = genetic advance; GAM (%) = genetic advance as percent of mean. CD (5%) = critical difference at 5% level of significance.

**Table 3:** Pollen morphometric parameters and pore density of the ten coconut varieties observed under scanning electron microscopy.

Variety	Polar Length (µm)	Pollen Width (µm)	(PW/PL) Ratio	Size Index	Pore Density (Pores/µm^2^)
Var 1	40.35 ± 1.99	34.19 ± 4.54	1.19 ± 0.11	13.80 ± 2.45	1.68 ± 0.83
Var 2	39.41 ± 4.02	31.46 ± 7.21	1.33 ± 0.48	12.40 ± 2.83	1.89 ± 0.60
Var 3	39.25 ± 2.37	35.22 ± 5.51	1.13 ± 0.11	13.82 ± 3.07	1.73 ± 0.50
Var 4	38.75 ± 1.83	36.32 ± 3.79	1.07 ± 0.09	14.07 ± 2.04	2.71 ± 0.47
Var 5	44.55 ± 4.67	40.15 ± 2.85	1.11 ± 0.06	17.89 ± 2.16	1.92 ± 0.85
Var 6	39.17 ± 3.84	31.56 ± 2.85	1.25 ± 0.23	12.36 ± 0.82	1.61 ± 0.30
Var 7	39.45 ± 2.76	28.71 ± 1.62	1.37 ± 0.09	11.33 ± 1.38	2.09 ± 0.68
Var 8	40.81 ± 4.56	37.27 ± 4.96	1.10 ± 0.11	15.21 ± 3.53	2.10 ± 0.29
Var 9	33.82 ± 4.18	30.80 ± 1.85	1.09 ± 0.08	10.42 ± 1.94	2.88 ± 0.28
Var 10	42.48 ± 1.28	40.07 ± 3.23	1.06 ± 0.07	17.02 ± 1.81	1.35 ± 0.69
Mean	39.80	34.58	1.17	13.83	2.00
CV (%)	8.42%	11.85%	12.60%	15.48%	26.15%
CD (*p* = 0.05)	3.42	4.15	0.18	2.48	0.61

Note: Values are presented as mean ± standard deviation (SD). Mean values are overall means across the evaluated varieties. CV: coefficient of variation; CD: critical difference at *p* = 0.05.

**Table 4:** Observed pollen shape classes and exine surface characteristics of the ten coconut varieties examined by scanning electron microscopy.

Varieties	Shape Classes	Aperture Type	Exine Ornamentation (at 3.0–10.0 K×)
Var1	navicular(dominant) + subglobose (rare)	Monosulcate (single distal sulcus)	Tectate-rugulate to foveolate-granulate with low irregular ridges (0.4–1.2 μm) and micro-perforations
Var2	navicular + occasional ovoid	Monosulcate	Finely reticulate-perforate with distinct lumina and thin muri
Var3	boat-shaped + subglobose in anther locule	Monosulcate	Tectate-rugulate-foveolate with granular supratectal elements
Var4	reniform + rounded minority	Monosulcate	Finely reticulate-perforate with shallow foveolae
Var5	navicular + subglobose	Monosulcate	Tectate-rugulate-granulate with additional subtle gemmate projections
Var6	navicular + scattered ovoid grains	Monosulcate	Punctate-foveolate with elevated ridges and prominent micro-pores
Var7	navicular (dominant) + minimal-collapse ovoid	Monosulcate	Tectate-rugulate-foveolate-granulate with gemmate elements
Var8	reniform + rare, rounded forms	Monosulcate	Finely reticulate-perforate with irregular muri
Var9	navicular + subglobose minority	Monosulcate	Finely reticulate-perforate with dense small lumina
Var10	navicular + ovoid	Monosulcate	Punctate-foveolate with distinct elevated ridges and micro-pores

## Data Availability

The original contributions presented in this study are included in the article. Further inquiries can be directed to the corresponding author.

## Supplementary Materials

The following supporting information can be downloaded at https://www.mdpi.com/article/10.3390/plants15182860/s1, Table S1. Results of normality and homogeneity of variance tests and the corresponding statistical procedures applied to the evaluated morphological, reproductive, pollen, and fruit-related traits. Table S2. Mean ± standard deviation and coefficient of variation (CV) for the morpho-agronomic, reproductive, fruit-related, and quality traits of the ten coconut varieties. Table S3. Description of the ten coconut varieties, including variety code, stature class, local name, sampling location, palm age, and number of sampled palms.
