# Supplementary material for: Integrative Morphological and Palynological Characterization of Cocos nucifera L.: Insights from Macro-Traits and Pollen Ultrastructure
Source: Plants (Basel). 2026 Sep 18;15(18):2860. doi: 10.3390/plants15182860 (PMC13610476; doi:10.3390/plants15182860)
Supplement: Supplementary file 1 [file plants-15-02860-s001.zip › plants-4532373-supplementary.pdf]

Supplementary Table S1. Results of normality and homogeneity of variance tests and the corresponding statistical procedures applied to the evaluated morphological, reproductive, pollen, and fruit-related traits.

Trait	Shapiro_W	Shapiro_p	Normality	Levene_F	Levene_p	Homogeneity	Recommended_Test
PL	0.966	0.4369	No significant departure	0.2859	0.9709	Equal variances	ANOVA + Tukey HSD
PW	0.9732	0.6308	No significant departure	0.2991	0.9664	Equal variances	ANOVA + Tukey HSD
PW.PL	0.8764	0.0023	Significant departure	0.5702	0.8055	Equal variances	Transformation / Kruskal-Wallis
SI	0.9718	0.59	No significant departure	0.3176	0.9595	Equal variances	ANOVA + Tukey HSD
PH	0.9556	0.2377	No significant departure	1.0007	0.4706	Equal variances	ANOVA + Tukey HSD
SG	0.9473	0.1428	No significant departure	0.4154	0.9117	Equal variances	ANOVA + Tukey HSD
NFF	0.9581	0.2765	No significant departure	0.7323	0.6755	Equal variances	ANOVA + Tukey HSD
IL	0.9478	0.1475	No significant departure	0.893	0.5485	Equal variances	ANOVA + Tukey HSD
NBP	0.9377	0.0788	No significant departure	0.1565	0.9964	Equal variances	ANOVA + Tukey HSD
NFB	0.9669	0.4571	No significant departure	0.2599	0.9787	Equal variances	ANOVA + Tukey HSD
TML	0.8653	0.0013	Significant departure	1.8375	0.1233	Equal variances	Transformation / Kruskal-Wallis
LFL	0.9548	0.2274	No significant departure	0.5997	0.7825	Equal variances	ANOVA + Tukey HSD
LLW	0.9434	0.1122	No significant departure	1.5014	0.2143	Equal variances	ANOVA + Tukey HSD
NLL	0.9801	0.8275	No significant departure	0.4898	0.8644	Equal variances	ANOVA + Tukey HSD
PLL	0.9753	0.693	No significant departure	0.2779	0.9734	Equal variances	ANOVA + Tukey HSD
LLS	0.9715	0.5812	No significant departure	0.5587	0.8143	Equal variances	ANOVA + Tukey HSD
LSS	0.9746	0.6714	No significant departure	0.7102	0.6935	Equal variances	ANOVA + Tukey HSD
SW	0.9639	0.3878	No significant departure	0.6733	0.7235	Equal variances	ANOVA + Tukey HSD
FW	0.9539	0.2142	No significant departure	0.7483	0.6625	Equal variances	ANOVA + Tukey HSD
HS	0.9451	0.1249	No significant departure	0.3257	0.9563	Equal variances	ANOVA + Tukey HSD
TSS	0.9586	0.2857	No significant departure	0.4978	0.8588	Equal variances	ANOVA + Tukey HSD

Supplementary Table S2. Mean ± standard deviation and coefficient of variation (CV) for the morpho-agronomic, reproductive, fruit-related, and quality traits of the ten coconut varieties.

Variety	PH	SG	NFF	IL	NBP	NFB	TML	LFL	LLW	NLL	PLL	LLS	LSS	SW	FW	FHS	TSS
Var 1	173.00±3.12	140.07±1.85	11.67±6.02	79.00±11.23	5.00±2.65	11.00±1.73	493.07±6.45	97.43±2.15	4.30±1.50	194.67±1.52	111.57±2.08	4.77±1.00	5.83±1.36	20.77±5.23	2683.33±150.20	4.73±0.42	5.10±1.04
Var 2	157.30±8.14	124.00±5.12	11.33±2.08	62.13±1.65	5.67±1.53	12.67±1.53	441.23±3.84	88.67±3.42	4.07±0.25	198.33±2.08	106.07±4.16	4.33±0.58	5.40±0.44	19.57±0.95	1742.33±112.50	3.93±0.72	4.93±1.12
Var 3	149.87±6.54	107.57±4.21	14.33±2.52	65.43±3.21	6.00±1.00	13.33±2.31	438.93±1.52	96.10±5.04	4.33±0.58	196.67±6.03	106.57±3.21	3.23±0.25	4.43±0.23	25.80±2.45	1571.67±88.40	3.57±0.29	5.07±1.50
Var 4	134.73±6.12	110.60±1.23	10.33±2.89	61.13±1.98	4.67±1.15	6.67±1.15	425.50±1.23	95.00±8.65	5.13±0.35	184.33±1.52	103.80±4.12	3.23±0.35	5.67±0.40	15.07±0.45	1604.67±95.20	3.47±0.29	5.53±0.74
Var 5	106.23±2.14	95.13±5.41	10.00±2.00	61.43±0.98	5.33±1.53	4.67±1.53	415.03±4.12	91.53±3.62	4.03±0.15	190.33±4.04	105.90±3.21	3.17±0.21	6.60±0.56	13.93±0.52	1683.00±102.40	3.53±0.32	5.57±0.67
Var 6	89.33±20.15	91.23±6.21	8.00±2.00	65.33±3.05	3.33±1.53	5.67±2.52	382.90±5.12	87.37±4.31	3.83±0.25	194.67±8.14	101.67±2.08	3.33±0.21	4.23±1.00	13.20±1.21	1495.00±75.60	3.83±0.29	5.67±0.67
Var 7	96.30±14.23	84.90±5.32	6.67±1.15	61.50±1.04	4.33±1.53	6.00±1.00	385.93±4.21	89.40±3.01	3.57±0.55	177.33±7.21	98.00±3.61	3.10±0.30	4.53±0.40	13.30±1.12	1275.00±62.30	3.67±0.12	5.10±0.10
Var 8	139.07±5.21	106.50±1.45	6.00±1.00	62.53±6.12	5.67±2.08	8.00±2.00	406.00±2.14	80.37±12.1	4.43±1.05	192.67±4.12	103.63±2.14	3.17±0.35	5.17±1.02	17.27±3.91	1833.67±110.50	4.20±0.30	3.97±0.15
Var 9	107.10±5.12	111.20±2.34	5.67±2.31	66.97±8.65	5.00±1.73	6.67±1.53	456.97±3.21	77.40±3.42	4.07±0.57	195.33±1.15	113.57±6.12	3.77±0.49	4.20±1.32	14.30±3.12	1775.67±98.40	4.37±0.32	4.30±0.26
Var 10	267.00±1.52	148.73±3.12	13.67±1.52	86.80±4.45	7.33±1.53	9.33±3.06	583.40±7.21	101.03±2.14	5.33±0.25	206.00±1.15	131.53±6.21	4.03±0.50	7.30±1.52	27.10±2.12	2994.00±165.40	5.57±0.15	5.30±0.25
CV (%)	36.60	18.31	31.68	12.93	20.38	36.24	13.41	8.24	12.71	4.05	8.62	16.19	19.58	28.54	29.03	16.17	10.83

Supplementary Table S3. Description of the ten coconut varieties, including variety code, stature class, local name, sampling location, palm age, and number of sampled palms.

Variety	Variety code	Stature class	Local name	Location	Age of Plam	No. of Sampled palms
Variety1	Var1	Tall	Wangwiset	Trang province	8	3
Variety2	Var2	Dwarf	Banphaeo	Trang province	7	3
Variety3	Var3	Dwarf	Banphaeo	Trang province	8	3
Variety4	Var4	Dwarf	Banphaeo	Trang province	8	3
Variety5	Var5	Dwarf	Banphaeo	Trang province	8	3
Variety6	Var6	Dwarf	Banphaeo	Trang province	8	3
Variety7	Var7	Dwarf	Banphaeo	Trang province	8	3
Variety8	Var8	Dwarf	Banphaeo	Trang province	8	3
Variety9	Var9	Dwarf	Banphaeo	Trang province	8	3
Variety10	Var10	Tall	Wangwiset	Trang province	9	3
